# Diet Quality Differs by Race/Ethnicity Among Mothers and Their Children from Supplemental Nutrition Assistance Program–Education Households

**DOI:** 10.1089/heq.2021.0007

**Published:** 2021-09-15

**Authors:** Fred Molitor, Celeste Doerr

**Affiliations:** ^1^ Department of Communication Studies, California State University Sacramento, Sacramento, California, USA.; ^2^ Public Health Institute Center for Wellness and Nutrition, Sacramento, California, USA.

**Keywords:** SNAP-Ed, diet quality, racial/ethnic disparities, mothers and children

## Abstract

**Purpose:** To investigate diet quality by race/ethnicity among mothers and their children from low-income households throughout California.

**Methods:** Cross-sectional telephone surveys of mothers and their children from randomly sampled households using a validated 24-h dietary assessment. Healthy Eating Index-2015 (HEI-2015) scores were calculated.

**Results:** The mean HEI-2015 scores were lower for African American and white mothers and children compared with Latinx mothers and children.

**Conclusion:** Addressing poor levels of overall diet quality among African American and white mothers and children from low-income households is of public health importance. Reasons for Latinxs' superior diet quality may include limited acculturation to U.S. culture.

## Introduction

The Healthy Eating Index 2015 (HEI-2015) is a measure of diet quality consistent with the recommendations of the 2015–2020 Dietary Guidelines for Americans (DGA).^1^ Higher HEI scores equate to better overall diet quality^2^ and are related to a reduced risk of obesity^3^ as well as cardiovascular disease, cancer, and all-cause mortality.^4,5^

Lower income is related to poorer diet quality.^3,6^ African American adults^3,6^ and children^6^ have been reported to have lower HEI scores than Latinxs and/or whites. Whether racial/ethnic disparities in diet quality exist within lower income populations is absent from the research literature. The objective of the current study was to investigate HEI-2015 scores by racial/ethnic groups among mothers and their children from low-income households throughout California.

## Methods

The study objective was addressed with data from 2018 to 2019 cross-sectional surveys of mothers and their children from households eligible for the Supplemental Nutrition Assistance Program–Education (SNAP-Ed). The data are from the most recent years of annual surveys implemented to obtain population-based SNAP-Ed indicators of dietary behaviors.^7^ The protocols for each survey year were approved by the California Health and Human Services Agency, Committee for the Protection of Human Subjects.

Households with reported incomes ≤185% of the federal poverty level and one adult female and a child(ren) were selected at random. The 2019 sampling frame excluded households recruited for the 2018 survey. In each survey year, a study introduction letter in English or Spanish was sent to the sampled households. Next, bilingual staff verified household eligibility by phone, and the names of the youngest mother (or caregiver) of children and one child 5 to 11 years, selected at random in multi-child households.

During the subsequent interviews, bilingual staff administered the Automated Self-Administered 24-H Dietary Assessment Tool (ASA24)^8^ in English or Spanish. The quantity and size of each reported food and beverage item were determined by asking mothers to reference pictures in a portion-size booklet or measuring cups or spoons that had been previously mailed to each household. The procedures were replicated to obtain 24-h dietary information for children.

Dietary information for mothers and their children was entered directly into the Web-based ASA24 system; responses to demographic items and the outcome of each recruitment call were entered into a separate database. Mothers and children were each sent a $15 gift card upon completion of the interviews.

Mothers and children were asked, “(Are you/is CHILD'S NAME) Hispanic, (Latina/Latino), or of Spanish origin?” The following question was, “What is (your/CHILD'S NAME) race? You may answer more than one. (Are you/is CHILD'S NAME) American Indian or Alaska Native, Asian, Black or African American, Native Hawaiian or other Pacific Islander, White, or Other. Mothers' age was ascertained by asking, “What is your age?” Children's age and gender were recorded from mothers during the study screening procedures. Mothers were asked, “What is the highest level of school you have completed or the highest degree you have received?”

HEI-2015 scores (theoretical range=0–100) were calculated based on established National Cancer Institute procedures^9^ from 13 components of dietary intake: total fruits, whole fruits, total vegetables, dark green vegetables and legumes, whole grains, total dairy, total protein foods, seafood and plant proteins, refined grains, added sugars, fatty acids, sodium, and total saturated fats.

Analyses for mothers and children were conducted separately. The mean HEI-2015 scores and 95% confidence intervals were calculated for the sample overall, and for Latinxs, African Americans, whites, and respondents coded as Other or Missing. Linear regression models were developed for HEI-2015 scores and the independent variable of race/ethnicity dummy coded with Latinx the reference group for African Americans, whites, and Other or Missing (each coded as 1 vs. 0). The regression models controlled for age (centered on the mean values), education for mothers, and gender for children. As covariates, mothers who reported education of 12th grade or less with no diploma were combined with missing and refused responses as the reference group for high school graduate/General Educational Development/and vocational, trade, or business school (1 vs. 0); or some college to a postgraduate or professional degree (1 vs. 0). Female children were coded as 1 and male children as 0. Number of recruitment attempts leading to and including the two completed household interviews was included as a covariate to account for the potential that survey participants who were more difficult to recruit may have had different dietary behaviors. Finally, whether the 24-h dietary recall period occurred on a weekend versus weekday was statistically controlled because Latinxs and whites have reported poorer levels of diet quality on weekends.^10^ The criterion for statistical significance was *p*≤0.05.

Excluded from the analyses were records for 105 mothers and 24 children with partial ASA24 interviews. A further 360 records for mothers were omitted due to implausible kilocalorie intake (≤400 or ≥4400 for adult women) per ASA24 procedures.^8^ Finally, 569 records for mothers whose child was not interviewed were omitted. Data merging, cleaning, coding, and analyses were conducted with SPSS (version 26.0; IBM Corp., Armonk, NY, 2019).

## Results

Complete and paired ASA24 interviews were available for 4166 mothers and children. Table 1 displays the demographics for the samples. The mean for number of recruitment calls to completed mothers' and children's interviews was 4.6 (range=1–22; standard deviation=2.8). More than one third of mother (36.9%) and children (37.7%) interviews required recall of weekend versus weekday 24-h food and beverage consumption.

**Table 1.** Characteristics of Sampled Mothers and Children from Supplemental Nutrition Assistance Program–Education Eligible Households Across California, 2018 and 2019 (*n*=4166)

Characteristic	Mothers	Children
Race/ethnicity, %		
Latinx	63.1	67.5
African American	12.6	13.4
White	19.0	14.4
Other	4.8	4.0
Missing	0.5	0.7
Female, %	100	49.7
Age, years		
Mean	35.4	8.1
Median	34.0	8.0
SD	7.7	2.0
Highest grade completed, %		
≤Eighth grade	10.5	—
Some high school or high school graduate	39.4	—
Vocational, business school, or some college	36.3	—
College or postgraduate degree	13.6	—
Missing	0.2	—

SD, standard deviation.

Unadjusted mean HEI-2015 scores for all mothers was 56.4 (95% confidence interval [CI] = 56.0–56.8) and for all children was 55.3 (95% CI = 54.9–55.7). The adjusted linear regression analyses found Latina mothers to report diets of higher quality than African American (*p*<0.001) and white (*p*<0.001) mothers (Table 2). Latinx children had significantly greater HEI-2015 scores than their African American and white counterparts (both *p*<0.001) as well as compared with children with Other or Missing race/ethnicity (*p*=0.044).

**Table 2.** Healthy Eating Index-2015 Scores by Race/Ethnicity Among Mothers and Children from Supplemental Nutrition Assistance Program–Education Eligible Households Across California, 2018 and 2019

	Mothers				Children			
	*n*	Unadjusted mean (95% CI)	Adjusted mean (95% CI)	*p*	*n*	Unadjusted mean (95% CI)	Adjusted mean (95% CI)	*p*
Latinx	2630	57.8 (57.3–58.4)	59.7 (58.6–60.8)		2812	56.1 (55.7–56.6)	56.3 (55.3–57.2)	
African American	526	53.1 (51.9–54.3)	55.3 (54.0–56.7)	<0.001	560	53.2 (52.1–54.3)	53.3 (52.1–54.5)	<0.001
White	793	53.9 (52.9–54.9)	56.0 (54.9–57.1)	<0.001	598	53.6 (52.5–54.6)	53.6 (52.4–54.8)	<0.001
Other or Missing	217	55.7 (53.8–57.6)	57.8 (55.9–59.7)	0.054	196	54.3 (52.3–56.4)	54.3 (52.3–56.2)	0.044

Regression analyses adjusted for age centered on mean, education for mothers, gender for children, the number of calls to completed interviews with mothers and children, and interviews occurring on weekends.

CI, confidence interval.

## Discussion

More than 11.8 million individuals in California, or one third of the state's population, are eligible for SNAP-Ed.^11^ Among mothers and children from these SNAP-Ed eligible households, we found that African Americans and whites were less adherent to the DGA,^1^ as operationalized by HEI-2015 scores, than Latinxs. Similarities in findings for mothers and their children are logical since mothers are often the gatekeeper of grocery purchases and meal preparation in households with children.^12^

Interpretation of our findings with those from the National Health and Nutrition Examination Survey (NHANES) suggests that California SNAP-Ed Latina mothers may have diet quality comparable to or better than the general U.S. female population. The mean HEI-2015 score for women participating in the 2011–2012 NHANES (59.7±0.6 [standard error])^13^ was significantly higher than the mean for our SNAP-Ed African American (55.3±0.67) and white (56.0±0.58) mothers, but equivalent to our Latina survey participants (59.7±0.56). The mean HEI-2015 scores for California SNAP-Ed Latina mothers exceeded those calculated for women participating in 2011–2014 NHANES,^14^ even for those classified as food secure (54.6±0.6). However, each of these scores receives a grade of “F” for adherence to the DGA,^1^ based on the graded approach proposed by Krebs-Smith et al.^15^ for interpreting HEI-2015 scores, with Latina mothers approaching a “D” grade (range=60–69).

The higher HEI-2015 scores for Latina mothers from SNAP-Ed eligible households in California may be due to protective cultural influences from incomplete acculturation to the dietary patterns typical of U.S. women. In studies of Mexican Americans participating in the NHANES, speaking Spanish at home was used in part or whole as a proxy for acculturation. In one study, acculturation was negatively related to HEI-scores.^16^ In another study, Spanish-only speaking adults, compared with adults who spoke Spanish and English equally, more English than Spanish, and only English, reported consuming fewer calories from fast food and sit-down restaurants, and eating more homemade meals.^17^ While the current study did not assess levels of use of Spanish versus English at home, preference to communicate in Spanish with bilingual interview staff was documented for 55.0% of mothers identifying as Latina. Subsequent analyses revealed that Latina mothers interviewed in Spanish had higher HEI-2015 scores than Latina mothers interviewed in English (60.9 vs. 55.0, *t*=11.3; *p*<0.001). As such, culturally influenced choices related to food purchasing and meal preparation may have resulted in the consumption of more healthful foods and nutrients, as assessed by the 13 HEI-2015 components, among SNAP-Ed Latina mothers, which when combined produced overall HEI-2015 scores well above those calculated for the more Americanized non-Latina, African American, and white mothers.

Limitations to the study include self-reported dietary information. The 24-h recall period of the ASA24 interviews may not have represented respondents' typical eating and drinking behaviors. Finally, inferences of the study findings should be made with caution, especially to populations that may not include Latinx adults and children with a preference to speak Spanish, which can be understood as a proxy for acculturation.

In conclusion, SNAP-Ed and other interventions designed to improve diet quality in California and possibly other low-income populations should continue to focus on mothers, and specifically on mothers identifying as African American and white. However, it should be understood that Latinx Americans' diets may be superior to those of their counterparts of other races, and that, based on past research, culture might be a protective factor.

## Abbreviations Used

ASA24: Automated Self-Administered 24-H Dietary Assessment Tool
DGA: Dietary Guidelines for Americans
HEI-2015: Healthy Eating Index-2015
NHANES: National Health and Nutrition Examination Survey
SNAP-Ed: Supplemental Nutrition Assistance Program–Education
